# Supplementary material for: Transcriptomic Analysis of Organotypic Porcine Retina Cultures
Source: Int J Mol Sci. 2026 Apr 28;27(9):3901. doi: 10.3390/ijms27093901 (PMC13163581; doi:10.3390/ijms27093901)
Supplement: Supplementary file 1 [file ijms-27-03901-s001.zip › ijms-4171478-supplementary file.pdf]

Supplementary materials

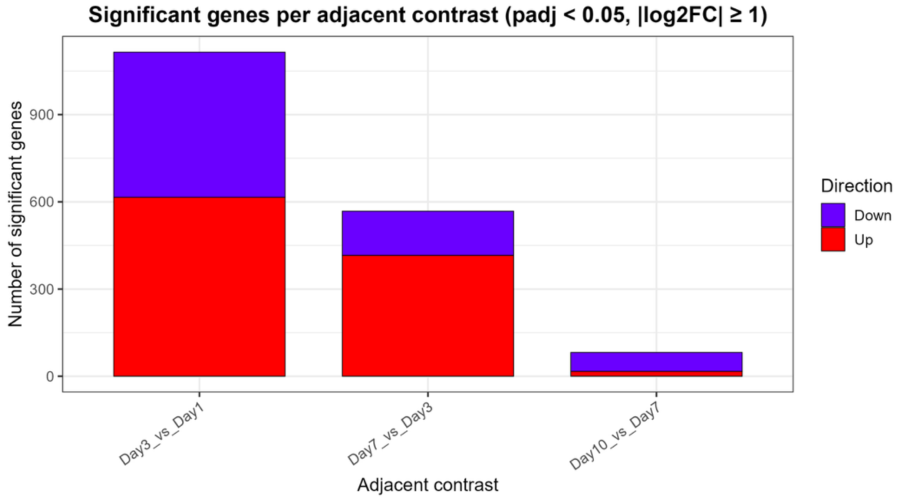

Supplementary Figure S1. The number of significant genes in adjacent contrasts decreases during the culture period. Related to Figure 2A.

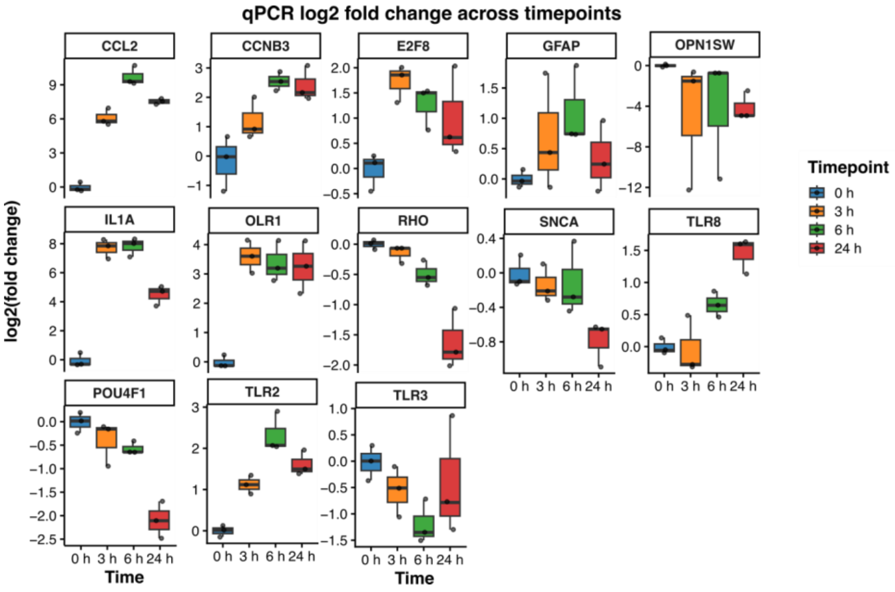

Supplementary Figure S2. mRNA levels of genes alter differentially in the first 24 hours after dissections. Related to Figure 2.

mRNA levels of selected genes were measured at 1, 3, 6, and 24 hours post-dissection.

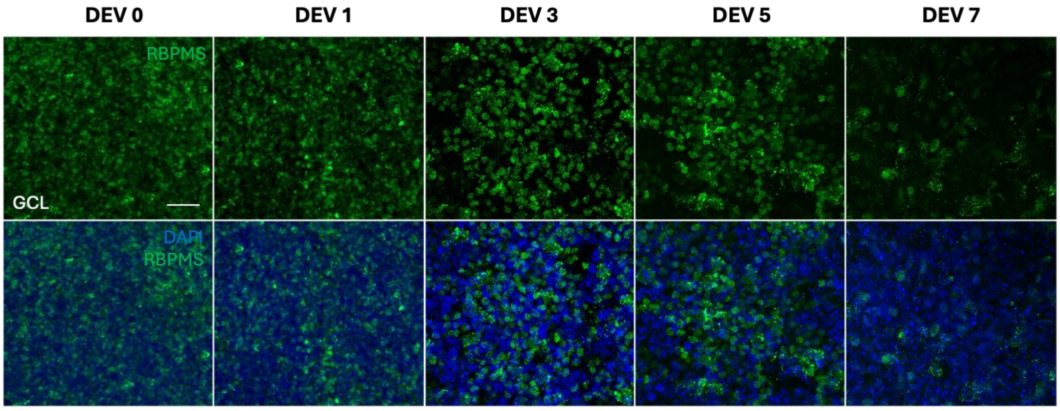

Supplementary Figure S3. Number of retinal ganglion cells reduces during the culture time. Related to Figure 5.

Porcine retina flat mounts were fixed, stained with anti-RBPMS antibody and DAPI, and analyzed via microscopy. The top panel displays RBPMS staining; the lower panel shows both DAPI and RBPMS staining (DEV = Day Ex Vivo).

Supplementary Table S1. Antibodies used in this study listed in staining order

Marker	Cat#, Vendor	Concentration or dilution	Antigen retrieval / antibody stripping	Fluorescent reagent
Ki67	ab16667, abcam	0.31 μg/ml	ER1 (pH6, 95°C/ 30 min)	OPAL 690
GFAP	ab7260, Abcam	1:8000	ER2 (pH9, 95°C/ 20 min)	OPAL 570
Iba1	019-19741, Wako	0.5 μg/ml	ER2 (pH9, 95°C/ 20 min)	OPAL 520
RBPMS	NBP2-20112- NovusBio	1 to250	none	

Supplementary Table S2. Gene probes (Thermo Fisher Scientific) for qPCR

Gene Symbol	Assay ID
GAPDH	Ss03375629_u1
GFAP	Ss03373547_m1
CCL2 / MCP1	Ss03394377_m1
POU4F1	Ss02916785_m1
OPN1SW	Ss03393999_m1
SNCA	Ss03379644_u1
RHO	Ss03394397_m1
IL1A	Ss03391335_m1
E2F8	Ss06897499_s1
CCNB3	Ss06916241_m1

TLR8	Ss03383235_u1
TLR2	Ss03381278_u1
TLR3	Ss03388861_m1
OLR1	Ss03392357_m1

Material and methods:

Porcine retinal explant preparation and immunohistochemistry:

Porcine eyes were surface-sterilized by immersion in 70% ethanol and dissected under sterile conditions. The anterior segment and vitreous were removed, and the posterior eye cup was flattened by four radial incisions. Retinal punches of 4 mm in diameter were obtained using a biopsy corer, avoiding the visual streak region to ensure tissue homogeneity. Individual explants were placed onto semipermeable membrane inserts in 6-well plates (ganglion cell layer facing upward) and cultured in Neurobasal-A medium supplemented with B27 (2%), GlutaMAX (1%), and Antibiotic-Antimycotic (1%) at 37°C and 5% CO_2. At designated time points day 0, 1, 3, 5, and 7, explants were fixed with 4% paraformaldehyde for 1 h at room temperature and processed as whole-mount preparations. For immunofluorescence, explants were permeabilized and blocked in 2% BSA / 2% Triton X-100 in PBS for 1 h at room temperature, then incubated with a rabbit anti-RBPMS primary antibody (NovusBio, NBP2-20112; 1:250) in blocking buffer for 48 h at 4°C. After three washes in PBS, explants were incubated overnight at room temperature in the dark with a donkey anti-rabbit Alexa Fluor 488 secondary antibody (Molecular Probes, A21206; 1:1000) together with DAPI (1:1000). Following additional PBS washes, flatmounts were mounted with Aqua Poly/Mount and imaged.
